# Perovskites-Based Solar Cells: A Review of Recent Progress, Materials and Processing Methods

**DOI:** 10.3390/ma11050729

**Published:** 2018-05-04

**Authors:** Zhengqi Shi, Ahalapitiya H. Jayatissa

**Affiliations:** Nanotechnology and MEMS Laboratory, Department of Mechanical, Industrial and Manufacturing Engineering (MIME), University of Toledo, Toledo, OH 43606, USA; Zhengqi.Shi@rockets.utoledo.edu

**Keywords:** perovskite, photovoltaics, thin film, fabrication, stability

## Abstract

With the rapid increase of efficiency up to 22.1% during the past few years, hybrid organic-inorganic metal halide perovskite solar cells (PSCs) have become a research “hot spot” for many solar cell researchers. The perovskite materials show various advantages such as long carrier diffusion lengths, widely-tunable band gap with great light absorption potential. The low-cost fabrication techniques together with the high efficiency makes PSCs comparable with Si-based solar cells. But the drawbacks such as device instability, J-V hysteresis and lead toxicity reduce the further improvement and the future commercialization of PSCs. This review begins with the discussion of crystal and electronic structures of perovskite based on recent research findings. An evolution of PSCs is also analyzed with a greater detail of each component, device structures, major device fabrication methods and the performance of PSCs acquired by each method. The following part of this review is the discussion of major barriers on the pathway for the commercialization of PSCs. The effects of crystal structure, fabrication temperature, moisture, oxygen and UV towards the stability of PSCs are discussed. The stability of other components in the PSCs are also discussed. The lead toxicity and updated research progress on lead replacement are reviewed to understand the sustainability issues of PSCs. The origin of J-V hysteresis is also briefly discussed. Finally, this review provides a roadmap on the current needs and future research directions to address the main issues of PSCs.

## 1. Introduction

The organic-inorganic halide perovskite solar cells (PSCs) have attracted a great deal of attention of solar cell research community due to an incredible device efficiency improvement from 3.8% to 22.1% since 2009 [1,2]. The perovskite already gained much attention as a potential replacement of the silicon photovoltaic (PV) devices, which is still occupied the most dominant position in the current PV market, with record efficiency of about 26% [3]. This small gap of solar cell efficiency attracted recent attention especially from the researchers with experience in dye-sensitized solar cells (DSSCs) or organic solar cells because some materials can be used in both PSCs and organic solar cells. The structure of PSCs also origins from the device structure of DSSCs [1]. The perovskite materials have been demonstrated with largely tunable band gap (e.g., CH_3_NH_3_PbX_3_ has a band gap from 1.5 eV to 2.3 eV) [4] and great light absorption coefficient (higher than 10^4^ cm^−1^) [5,6], which is similar to other thin film solar cell materials such as CdTe [7] and copper zinc tin sulfide (CZTS) [8]. Its low-cost and convenient fabrication techniques also serve as the possible advantages over silicon-based devices that require complicated and costly high-vacuum deposition methods. Reports of successful cell fabrication on flexible substrates even indicated a greater possibility to the large-scale roll-to-roll manufacturing of PSCs that can be used in the industries [9,10,11].

The initial meaning of “perovskite” was about the crystal structure of calcium titanate, which was discovered in 1839 by the German mineralogist Gustav Rose and was named by the Russian mineralogist Lev Perovski. Since then, the term “perovskite” was referred to all compounds with the same crystal structure as calcium titanate. The perovskite light absorption layer has a general formula of ABX_3_, where A is an organic cation (e.g., methyl-ammonium CH_3_NH_3_^+^), B is a metal cation (e.g., Pb^2+^) and X stands for the halide anion (e.g., I^−^).

The first record of perovskite-based solar cell efficiency, however, was reported by Miyasaka et al. [1] only less than one decade ago. They reported an efficiency of 3.8% based on a DSSC structure. Due to the application of liquid electrolyte in the hole-transporting material (HTM), the stability of solar cell was very weak and did not attract much attention. Similar trial was done by Park et al. [12] with the increased efficiency of 6.5% but stability was still the main problem because of the instability of HTM layer due to the liquid medium.

**Figure 1:**
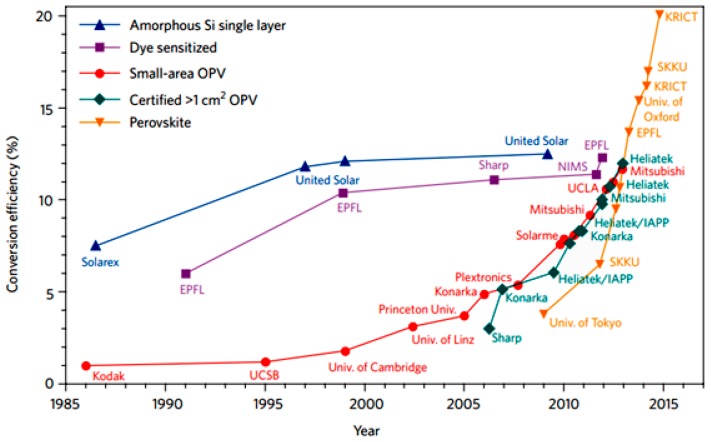
A comparison of perovskite efficiency progress with other kinds of photovoltaic (PV) devices (Reprinted with permission) [19].

The application of solid-state HTM (2,2′,7,7′-tetrakis(*N*,*N*-di-pmethoxyphenylamine) -9,9′-spirobifluorene, i.e., Spiro-OMeTAD), rather than liquid HTM, onto the highly-crystallized perovskite layer triggered the efficiency boosting during the past several years. Lee et al. [13] reported a breakthrough device efficiency of 10.9% in 2012 with the open-circuit voltage higher than 1.1 V. Wang et al. [14] introduced graphene into PSCs and acquired an efficiency of 15.6% in 2013 and the application of another perovskite material, formamidinium iodide (HC(NH_2_)_2_PbI_3_) together with poly-triarylamine (PTAA) as a new HTM brought a remarkable 20.1% efficiency in 2015 [15]. The current record efficiency of PSCs was 22.1%, created in 2016 by Seong Sik Shin et al. [16]. They also accomplished a long-term and stable efficiency of 21.2% in another work [17]. The perovskite-inserted tandem cell also achieved a promising efficiency of 26.7% by combining with Si cells [18]. During this progress, various HTM and vacuum/non-vacuum fabrication methods have been developed, which would be discussed later in this review. Figure 1 compared the efficiency progress of PSCs with other 3rd generation photovoltaics up to date [19]. The rapid improvement of the efficiency of PSCs make perovskite being expected to be comparable with the stable performance of c-Si solar cells whereas all other kinds of non-silicon solar cells suffered great barriers in further improvements. According to the theoretical calculation based on the well-known Shockley-Queisser limit, the perovskite devices, which have (CH_3_NH_3_PbI_3−x_Cl_x_), could achieve an efficiency around 25–27% [20]. This result indicates that there is still opportunity for the improvement of PSCs.

Although laboratory scale PSCs exhibited a great progress, perovskite-based PVs still needs to overcome several barriers. In general, there are two major problems currently blocking the improvement pathway: device instability of device performance [21,22] and hysteresis of J-V (current density-voltage) [23]. At present, long-term efficiency measurements (>1000 h) is still not adequate for the commercialization of PSCs. The PSCs must pass a series of testing under harsh conditions and environments for similar duration (>1000 h) [24]. Thus, it is very important to understand the degradation mechanism of both perovskite materials and other device components such as hole transport medium (HTM) and electron transport medium (ETM). The J-V hysteresis was discovered during cell testing when voltage sweeping routine changed. This phenomenon brings problems for standardizing the measurement protocol of PSCs. In addition, the toxicity from lead could be another problem during the manufacturing, using and recycling of perovskite [25]. Currently several trials on applying non-toxic alternative metal ions have been reported [26,27] but their device efficiency is still not promising. Detail information could be found later in this review.

Future research of PSCs, except efforts on improving the stability and reducing J-V hysteresis of PSCs, could also be focus on the large-area fabrication of PSCs (even small module area) and efforts on at least partial replacement of lead with other non-toxic metal ions inside the perovskite. Bi-facial illumination could also be considered for PSCs due to its structural advantages. Detail information could be found in the last part of this review.

It has been clear that the perovskite could be the next candidate to replace Si due to its outstanding structural, electrical and optical properties. This review, therefore, would start with the discussion from micro-scale observations on the crystal and electrical structures of perovskite materials. The next part is the discussions on device-level investigations: the evolution of device structure, the fabrication methods and their progresses and the exploration of each device component. We would then focus on the research efforts of device stability and toxicity of PSCs and finally show our suggestions for further directions of the perovskite research.

## 2. Structures

### 2.1. Crystal Structure

**Figure 2:**
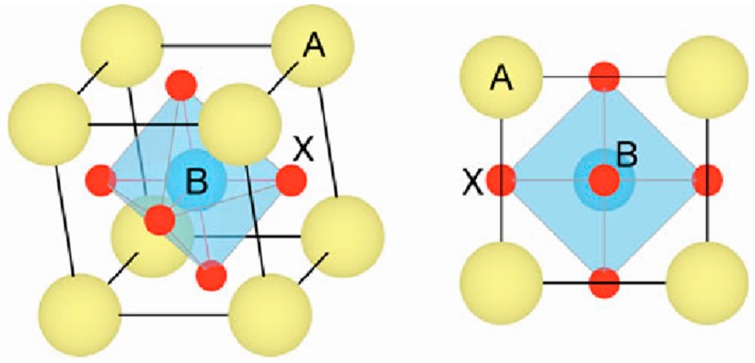
A generic perovskite crystal structure of the form ABX_3_ (Reprinted with permission) [31].

The perovskite materials have a general crystal structure described as ABX_3_, where “A” and “B” are cations with varied sizes and “X” is an anion. A typical unit cell structure of a basic perovskite compound is shown in Figure 2. Organometallic halide perovskites include an organic cation (e.g., methyl-ammonium CH_3_NH_3_^+^, ethyl-ammonium CH_3_CH_2_NH_3_^+^, formamidinium NH_2_CH=NH_2_^+^), a metal cation of carbon family (i.e., Ge^2+^, Sn^2+^, Pb^2+^) and a halogen anion (i.e., F^−^, Cl^−^, Br^−^, I^−^). Among them, methyl-ammonium-lead-iodide (MAPbI_3_) is the most widely used perovskite light absorber. Some recent research efforts also replaced lead with other metal ions due to the concern of toxicity of lead during device fabrication, especially for the future large-scale manufacturing [26,28]. In addition, several organic cations (CH_3_NH_3_^+^ and NH_2_CH=NH_2_^+^), inorganic cations (Cs^2+^ and Sn^2+^) and halide anions (Br^−^, Cl^−^ and I^−^) have been used to improve the efficiency and stability [29,30].

**Figure 3:**
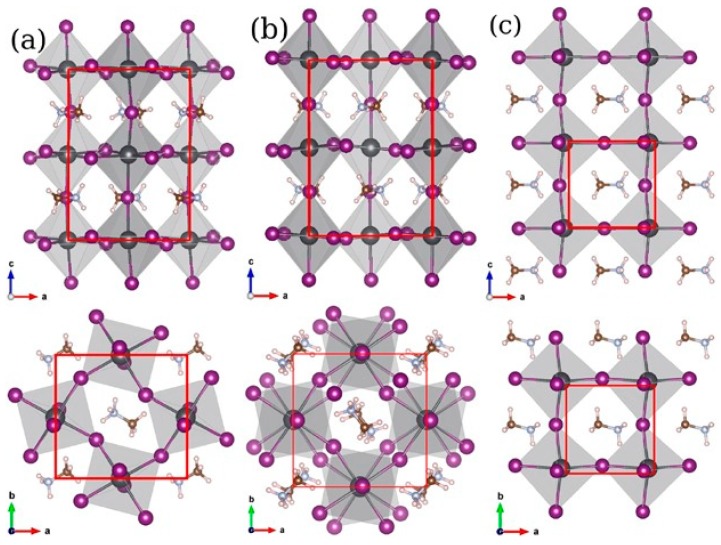
Comparison of (**a**) orthorhombic; (**b**) tetragonal and (**c**) cubic perovskite phases obtained from structural optimization of MAPbI_3_. Top row: a-c-plane and bottom row: a-b-plane (Reprinted with permission) [35].

Perovskite materials have different phases depending on the change of temperature. When temperature is lower than 100 K, the perovskite displayed a stable orthorhombic (γ) phase. With temperature increased to 160 K, the tetragonal (β) phase started to appear and replace the original orthorhombic (γ) phase [32]. As temperature increases further to about 330 K, the tetragonal (β) phase started being replaced by another stable cubic (α) phase [33]. Figure 3 displayed all those three crystal structures. The tetragonal-cubic phase transition at higher temperature partially influences the thermal stability of perovskite materials. Formamidinium iodide (HC(NH_2_)_2_PbI_3_), for example, has a phase transition occurred at a higher temperature, indicating that it is relatively stable compared with common MAPbI_3_. Moreover, a recent report suggested that light soaking could also trigger the reversible phase transition of perovskite materials [34] but more efforts are required to demonstrate this behavior.

### 2.2. Electronic Structures

**Figure 4:**
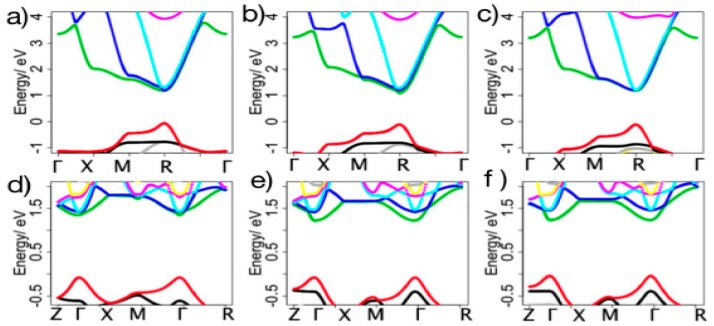
(**a**–**c**) showed the band structure of cubic MAPbI_3_ optimized with lead relaxed, corresponding to 001^−^, 110^−^ and 111^−^ MAPbI_3_, respectively. The relative results of tetragonal phase results are shown in (**d**–**f**) (Reprinted with permission) [36].

The electronic structure of perovskite, especially the typical MAPbI_3_, was already estimated by DFT (density functional theory) calculations. The calculated band gap had a good agreement with the measured band gap by absorption spectrum even after considering the spin orbit coupling and other interactions like van der Walls interaction. Zhou et al. [36] studied the band structure of both cubic and tetragonal MAPbI_3_ and the results were shown in Figure 4.

Also, the unusual DOS (density of state) position of Pb^2+^ and I^-^ showed the p-p optical transition, which was similar to the charge transition of an ionic material [37]. On the valence band maximum (VBM), due to the s-p antibonding coupling, the valence band top tends to dispersion, which leaded to a smaller effective mass (m_o_). According to other calculations [38,39,40], it is believed that MAPbI_3_ had an effective mass with the same magnitude of widely-used Si and GaAs. Thus, a high carrier mobility could be expected. Although further investigation did not match this estimation with the same magnitude [41], the evidence of low radiative recombination coefficient of MAPbI_3_ indicated the carrier mobility is high enough to overcome the radiative recombination [42]. Besides, long carrier lifetime and suitable diffusion length of MAPbI_3_ were estimated [43]. Compared with the long diffusion length of Si and GaAs (10^1^–10^2^ µm) [44,45], a shorter diffusion length (<10 µm) of polycrystalline thin film perovskite were interpreted as due to the grain boundary effects [46,47].

**Figure 5:**
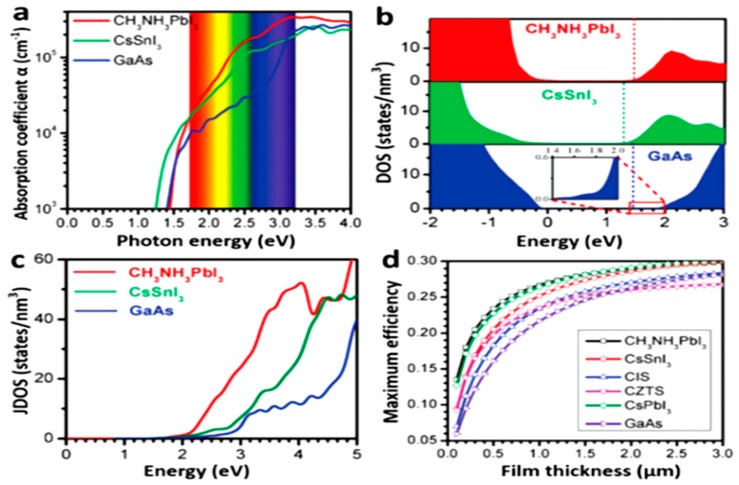
(**a**) The optical absorption, (**b**) Density of state (DOS) and (**c**) Joint density of states (JDOS) of MAPbI_3_, CsSnI_3_ and GaAs. (**d**) Calculated maximum efficiencies of MAPbI_3_, copper indium sulfide (CIS), CZTS and GaAs as a function of film thickness (Reprinted with permission) [48].

Moreover, by comparing the DOS and the absorption spectra of MAPbI_3_ and GaAs shown in Figure 5 [48], it could be concluded that the p-p transition is stronger than typical p-s transition in GaAs. The clear difference of DOS close to the conduction band minimum (CBM) led to the difference in joint density of states (JDOS) and therefore, generated the higher light absorption shown in Figure 5. Thus, the efficient charge generation and transition lead to a high photo-current and voltage with proper device structure.

### 2.3. Device Structure

**Figure 6:**
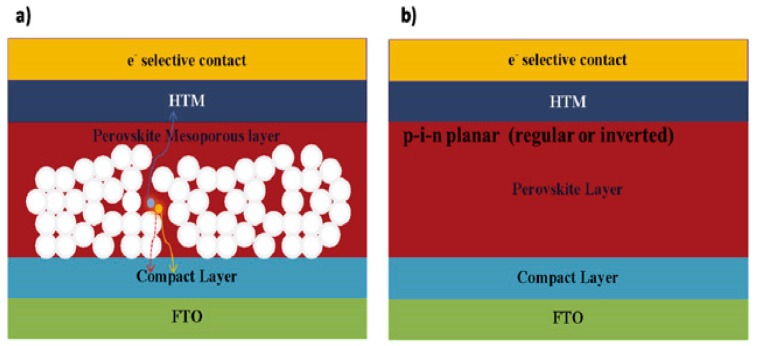
Schematic diagrams of (**a**) mesoscopic and (**b**) planar perovskite solar cells (PSCs) (Reprinted with permission) [52].

The first reported perovskite device is designed based on the structure of DSSCs, where liquid electrolyte capped both mesoporous TiO_2_ particles and perovskite material as the new “dye” molecules. Their work demonstrated the perovskite was not a stable “dye” due to its quick dissolving in the liquid hole-transport layer. The 3.1% and 3.8% device efficiency (depends on different halogen anions) could only last few minutes [1]. A later research used similar structure but thinner TiO_2_ layer (from 8–12 µm to 3 µm) and the efficiency increased to 6.5%. The perovskite was also proved a better light absorption than the dye molecules (N719); however, the corrosion appeared in liquid electrolyte and destroyed the device after 10 min [12]. To avoid this degradation, a solid-state hole-transport material was applied and the device performance was significantly increased. According to Lee et al. [13], this improvement combined both features from thin-film PVs and DSSCs and many other works were accomplished on increasing the efficiency. Solar cells were fabricated similar to thin-film PV, where perovskite served solely as the light absorber without TiO_2_ assistance. They finally acquired a planar PSC with a 1.8% efficiency [13]. They modified the growing condition of perovskite and boosted the efficiency to 11.4% but TiO_2_ was still the charge blocking layer [49]. At present, both planar and mesoscopic structure-based cells have efficiency of 20.8% [50] and 21.6% [51], respectively. A schematic of both planar and mesoscopic structure could be found in Figure 6 [51]. The PCSs could be fabricated in both sequences rather than thin-film PV, whose device configuration was limited by the properties of absorber materials. Thus, there are four major types of PSCs: substrate/superstrate-configured mesoporous structure and substrate/superstrate-configured planar structure.

The most typical n-i-p mesoporous structure is the first demonstrated high-efficient structure for perovskite devices. Started with the TCO cathode (mostly fluorine-doped tin oxide, FTO), a thin compact blocking layer was applied to decrease shunting, a mesoporous metal oxide layer filled with highly crystalline perovskite absorber layer. A layer of HTM was applied and a metal contact layer was deposited on the top of the device.

The mesoporous structure is originated from typical DSSCs. The reason for the weak performance of DSSC-based perovskite devices, except the corrosion due to liquid electrolyte mentioned above, was the excess mesoporous TiO_2_ part. The widely-spread TiO_2_ nano-particles inside the perovskite layer reduced the growth of perovskite crystals and also decreased the distance between separated free carriers, giving extra change for carrier recombination between TiO_2_ and HTM layer. Research results showed that the perovskite device acquired a higher efficiency with thinner mesoporous layer [12]. Therefore, in n-i-p mesoporous structure of PSCs, the mesoporous layer was normally less than 300 nm. Such structure allows perovskite to form a capping layer on top of the mesoporous part, serving as a light-sensitive intrinsic layer while reducing the carrier recombination process. Currently mesoporous structure is one of the most popular structures in the fabrication of PSCs with a power conversion efficiency (PCE) greater than 20% [50]. Other materials such as Al_2_O_3_ and ZrO_2_ have been also reported with great device efficiency [53].

The planar PSC is successful because it utilizes thin-film PV structure and excellent optical and electrical properties of perovskite. It is also an extreme case for mesoporous structure, where the thickness of mesoporous layer is zero and unlike the mesoporous structure, this type of structure could be fabricated without high-temperature process [52]. This structure requires a better control of the formation of perovskite absorber and suitable choice of HTM/ETM layers. Research efforts demonstrated a PCE of 21.6% for this type of PSC [51]. However, an ultra-thin mesoporous charge transport layer was always applied at the interface of perovskite and mesoporous TiO_2_ in order to enhance the carrier collection [15].

## 3. Fabrication Approaches

### 3.1. Perovskite Layer Fabrication

Because of the structural similarities of PSCs with both DSSCs and thin-film PVs, the fabrication approaches for both kinds of solar cells, including almost all vacuum and non-vacuum methods, could have a considerable improvement in PSCs as well. But the actual research showed something different: due to relatively easier process and great efficiency output, spin-coating is the most widely used method in the fabrication of PSCs but it is not suitable for large-scale manufacturing. Many other non-vacuum-based approaches were also developed and will be mentioned below. Some of them, such as doctor blading and screen printing, had been also successfully applied for the fabrication of larger-scale perovskite films [54]. However, thermal evaporation is the only vacuum-based methods that ever been demonstrated with a good cell performance. To the best of our knowledge, sputtering was never used possibly due to the lack of appropriate sputtering target and the possible damage of high-energy species to the unstable perovskite materials. According to different preparation procedures, the fabrication approaches of PSCs could be categorized as: one-step process; two-steps process; vapor-assisted process and thermal evaporation process.

#### 3.1.1. One-Step Method

One-step deposition was widely used in perovskite cell fabrication due to its easier operation and low cost. The perovskite film could be fabricated with pinhole-free and suitable stoichiometry with wise control of perovskite precursors. Typically, the perovskite precursor solution was prepared with organic halide (MAI/FAI, methylammonium/formamidinium iodide) and inorganic halide (e.g., PbI_2_) dissolved in gamma-butyrolactone (GBL), dimethylformamide (DMF), dimethyl sulfoxide (DMSO) or a combination of two or all three solvents. The mixed precursors were spin-coated and annealed in a range of 100–150 °C to form phase-pure, pinhole-free and dense perovskite layer.

**Figure 7:**
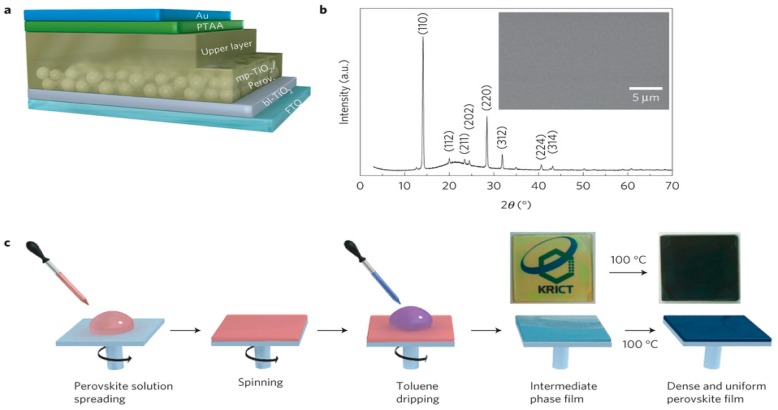
(**a**) Device structure of the bi-layered PSC; (**b**) X-ray diffraction (XRD) pattern of the annealed perovskite on fused silica. A surface scanning electron microscopy (SEM) image of fluorine-doped tin oxide (FTO)/bl-TiO_2_/bi-layered TiO_2_-perovskite composite is inserted; (**c**) one-step perovskite film fabrication steps (Reprinted with permission) [55].

One-step approaches had a great starting point of a 10.9% efficiency reported by Lee et al. [13], where the as-synthesized MAI and commercially-available PbCl_2_ were dissolved in DMF in a molar ratio of 3:1 in order to adjust the halide anion ratio. The perovskite layer formed after 30 s of spin coating and 100 °C post-annealing. The device also displayed a great open circuit voltage (V_oc_) of more than 1 V. Since then, various solution-based methods have been developed. One group found an intermediate state MAI·PbI_2_·DMSO, which could assist the formation of uniform and dense bi-layer perovskite absorber layer (mp-TiO_2_ with nano-scale MAPbI_3_/crystal MAPbI_3_) as shown in Figure 7 [55]. This phenomenon was followed with various solution adjustment that tried to form the desired intermediate state: Rong et al. [56] reported the formation of non-stoichiometric MA_2_Pb_3_I_8_(DMSO)_2_ in a DMSO/GBL mixed solvent (3:7 *v/v*) and they suggest this phase would assist with forming smooth perovskite layer. In addition, their work also discovered strong dependence of process conditions on the device performance: their PCE varied from 8.07% to 15.29% with different post-annealing temperature and time. Guo et al. [57] showed the formation of a PbI_2_–MAI–DMF complex in a temperature range of 40–80 °C. At temperatures higher than 100 °C, the prepared perovskite films displayed a better phase purity.

**Figure 8:**
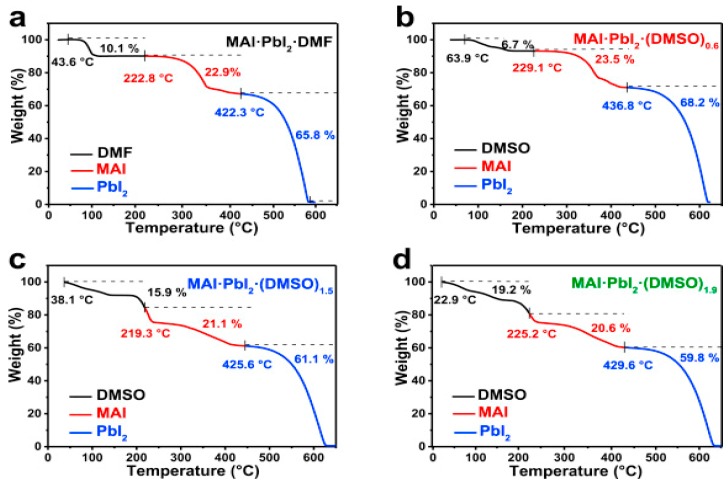
Thermogravimetric analysis (TGA) of (**a**) MAI·PbI_2_·DMF_x_ powder and (**b**–**d**) MAI·PbI_2_·(DMSO)_y_ (y = 0.6, 1.5, 1.9) powder. The black, red and blue solid lines indicate mass loss behavior of the DMF (DMSO), MAI and PbI_2_, respectively (Reprinted with permission) [59].

**Figure 9:**
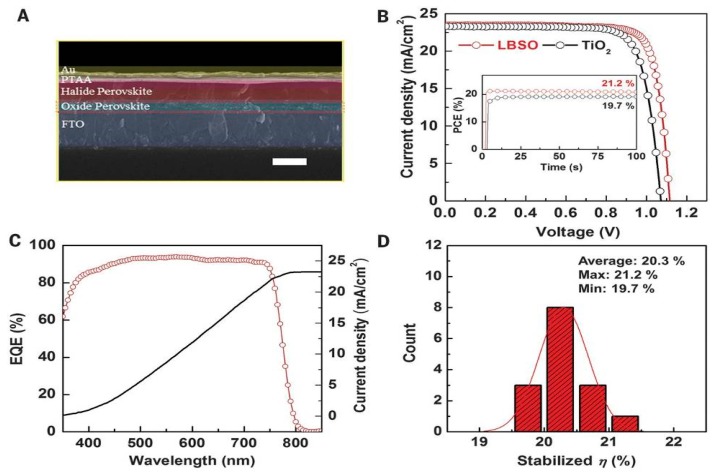
Photovoltaic performance of PSCs (**A**) Cross-sectional SEM image of LBSO-based PSCs (scale bar, 500 nm). (**B**) J-V curves and (inset) stabilized power conversion efficiencies (PCEs) at a maximum power point (LBSO: 0.96 V; TiO_2_: 0.91 V) for the best LBSO- and TiO_2_-based PSC. (**C**) External quantum efficiency (EQE) spectrum and J_sc_ integrated from the EQE spectrum of the best LBSO-based PSC. (**D**) Histograms of PCEs extracted from an I_sc_ stabilized at the maximum power point during 100 s for the LBSO-based PSCs (Reprinted with permission) [17].

The formation of one dimensional MA_3_PbI_9_(DMSO)_2_ and MA_3_PbI_9_ (DMSO) phases was also found, which brought a discussion of the perovskite formation mechanism. The results indicated that the DMSO was a better solvent [58]. Moreover, a controllable MAI·PbI_2_·(DMSO)_1.5_ was examined through TGA (thermogravimetry analysis) as shown in Figure 8 [59]. The perovskite was synthesized in a DMSO/DMF mixed solvent (85:15 *v/v*) and the final device showed a high short-circuit-current density (J_sc_) of 21.39 mA/cm^2^, a V_oc_ of 1.06 V, a fill factor (FF) of 0.76 and a PCE of 16.41% [59]. The current record of PSC (Figure 9), was a stable efficiency of 21.2% [17]. Except their replacement with Lanthanum (La)–doped BaSnO_3_ (LBSO) of the typical mesoporous TiO_2_ layer to increase the stability of perovskite, their precursor solution also included 2-Methoxyethanol, DMSO and GBL with the volume ratio of 7:3:4.

Another factor on one-step fabricated PSC is the additives applied after the precursor deposition. Liang et al. [60] first discovered a controllable perovskite crystallization rate with the application of 1,8-diiodooctane (DIO) to the precursor solution. They found that the additives reduced transformation kinetics and allow a homogeneous crystal growth. Thus, more pinhole-free perovskite crystals were produced hence the surface morphology and device performance were improved. A PCE of 11.8% was accomplished through this process. Since then, more additives were demonstrated enhancing the device efficiency, such as NH_4_Cl [61], HI [62] and CH_3_NH_3_Cl [63].

The proper adjustment of composition would also benefit the PCE of devices. Recently, a PCE of 20.26% was claimed by Nazeeruddin et al. [64]. Their achievement was accomplished not only due to the complex additive of Li-bis(trifluoromethanesulfonyl) imide, FK209 [tris(2-(1H-pyrazol-1-yl)-4-tert-butylpyridine)-cobalt(III) tris(bis(trifluoromethylsulfonyl) imide) and 4-tertbutylpyridine but also a different precursor preparation technique by starting with a mixture of MAI and FAI. Also, the band gap tuning of perovskite would be accomplished by either modifying the organic cations or adjusting halide anion ratios [65]. Corresponding device performance change had already been appeared in some reports with reproducible results [15,66,67].

Although spin-coating is used for the fabrication of PSC layers, successful devices processed on other non-vacuum based approaches had also been reported, including doctor-blade coating [68], spray coating [69], inkjet printing [70] and slot die coating [71]. All those approaches could be considered as alternative pathways for the fabrication of PSC. However, a general disadvantage of those methods is the poor control on perovskite surface morphology, which would highly affect the performance of PCE of PSCs.

#### 3.1.2. Two-Step Method

The perovskite deposition by two steps requires no complete precursor preparation but separate the coating of PbX_2_ (X=Cl, Br or I) and MAI/FAI layers. First, a PbX_2_ seed layer would be fabricated (spin-coating, doctor blading) on a substrate. Next, the MAI/FAI incorporation would be done by either dipping the PbX_2_-covered substrate into MAI/FAI solution (normally isopropanol) [72] or spin-coating of MAI/FAI [73] solution. The final perovskite films would be formed after proper baking. Although steps become more complicated, the morphology and quality of perovskite films could be better controlled via adjusting parameters in either step, which is more process-tunable than one-step fabrication.

**Figure 10:**
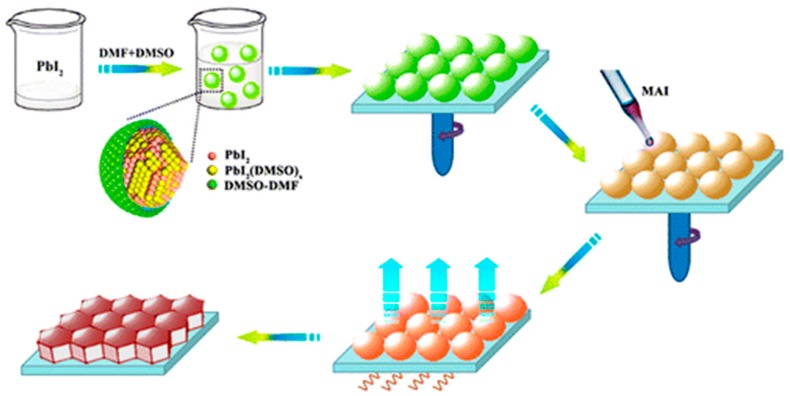
A schematic draw of the DMSO-assisted two-step MAPbI_3_ synthesis and film growth (Reprinted with permission) [76].

**Figure 11:**
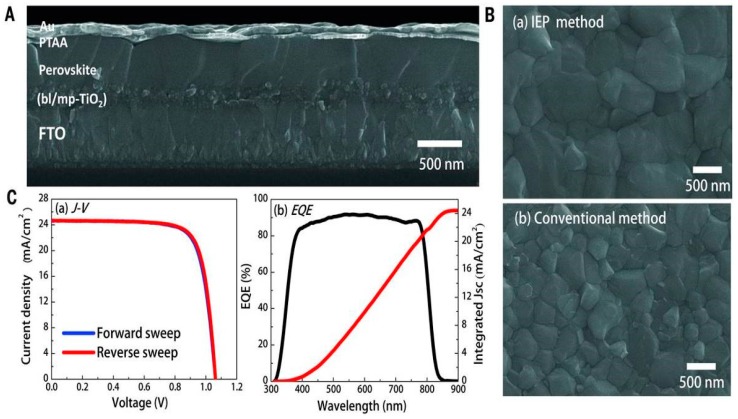
SEM observations and J-V and EQE measurements. (**A**) Cross-sectional FESEM image of the device; (**B**) The comparison of FESEM surface images of FAPbI3-based layer formed on mp-TiO_2_ by IEP and conventional method. (**C**) (**a**) J-V curves of best device measured with a 40-ms scanning delay in reverse (from 1.2 V to 0 V) and forward (from 0 V to 1.2 V) modes under AM 1.5G illumination and (**b**) EQE spectra for best device and integrated JSC (Reprinted with permission) [15].

In 1998, IBM [74] first synthesized perovskite on glass substrate. After 15 years, the Grätzel Group successfully fabricated the first perovskite cell that had 15% efficiency [75] by using this approach. Due to similar principles of one-step and two-step methods, proper solution engineering including solvent mixing and use of additives could be also applicable to two-step-fabricated PSCs: Li et al. [76] reported an improved PCE of 17.16% with mixing DMSO with DMF due to better coordination of DMSO with PbI_2_ and an extra intermolecular exchange between DMSO and MAI, which assisted the decomposition of intermediate state and the formation of perovskite. Figure 10 showed a schematic process draw to display the reaction between MAI and PbI_2_ at the outer solvent shell. Another work demonstrated the addition of a trace amount of H_2_O would also reduce voids and pinholes on the PbI_2_ precursor layer and generated an efficiency of 18% and a remarkable fill factor of 85% [77]. The record PSC using two-step method could already achieved a PCE of 20.2% by introducing PTAA and taking advantage of intramolecular exchange with DMSO catalysis [15]. The device shown in Figure 11 had little hysteresis effect and this new method assisted grain growth.

**Figure 12:**
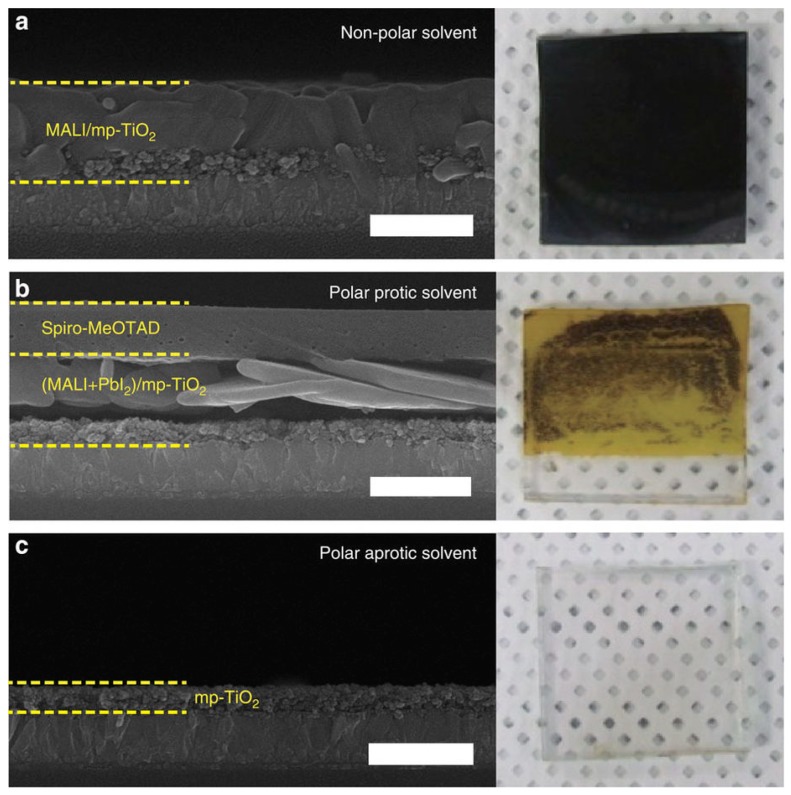
Cross-sectional FESEM and images of dissolved PCSs obtained after 30 s immersion of spiro-MeOTAD/MALI/mp-TiO_2_/TCG in (**a**) non-polar; (**b**) polar protic and (**c**) polar aprotic solvents. Diethyl ether, water and DMF, respectively, were used as the representative solvents. Scale bars, 700 nm (Reprinted with permission) [80].

Since two-step fabrication relies on a second MAI immersion/layer fabrication, the perovskite formation may not be as complete as in one single precursor solution. Someone considered that due to low temperature and short-time mixing (less than 1 min) during spin-coating, the diffusion of MAI into PbI_2_ lattice is not fast enough to form the perovskite crystals [78], or maybe only enough to form perovskite on the PbI_2_ surface, in which the perovskite layer blocked further diffusion of MAI to the inner part of PbI_2_ [79]. In general, the non-stoichiometry would have negative effect on the device efficiency. Another disadvantage comes from the partial dissolving of perovskite during the second step. Relevant researches have already proven that such mass migration speed in step two, as shown in Figure 12, could be very fast, depending on the properties of solvent [80]. The most direct result could be a rough surface with pinholes and voids, which could be easily formed during two-step process. This drawback could be resolved with addition of suitable chemical or use of low-concentration of MAI/FAI solution in order to improve the perovskite crystal growth condition.

#### 3.1.3. Vapor-Assisted Solution Method

**Figure 13:**
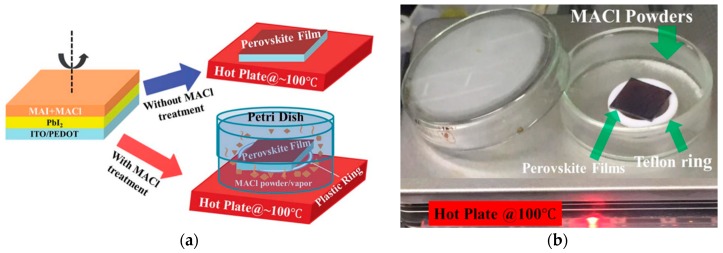
(**a**) Schematic drawing of vapor-assisted perovskite deposition process; (**b**) actual lab set-up mentioned by Khadka et al. [82] (Reprinted with permission).

Vapor-assisted solution method could be considered as a modified two-step method. During the second step, vaporized MAI/FAI reacted with PbI_2_ to form perovskite phase after further film annealing. Ideally, this approach could guarantee a better contact between both precursors than in the solution. Furthermore, this method successfully avoids the partial perovskite dissolving especially during the dipping process. Therefore, the perovskite film stoichiometry could be also improved. Chen et al. [81] developed this approach by using as-synthesized MAI vapor (very small particles) applied on spin-coated PbI_2_ precursor under a 150 °C baking. The whole perovskite fabrication was done in glove box. They reported µm-scale grain formation, full phase transition and film coverage. Their best planar device revealed a PCE of 12.1%. The only disadvantage was the key process lasted for hours, rather than minutes for spin-coating. This approach was later modified with an as-grown perovskite layer, where a two-step as-deposited MAPbCl_3−x_I_x_ on ITO/PEDOT:PSS substrate was then transferred into a closed petri dish container and heated together with MACl powder starting from 100 °C, which resulted a great PCE improvement of 15.1% with a 60-day stability [82]. Figure 13 offered a detail description about this process, where both upper and lower dish are linked with a Teflon ring to against the possible leakage. But other details such as the heat-treatment duration was not mentioned. Recently, a device with a planar structure of FTO/compact-TiO_2_/C_60_/(FA)_x_(MA)_1−x_PbI_3_/spiro-OMeTAD/Au was fabricated by heating FTO/c-TiO_2_/C_60_/PbI_2_ with uniformly-spread FAI and MAI powders in low vacuum under 170 °C for 30 min. By adjusting the powder ratio, they finally achieved a PCE of 16.48% [83]. The vapor-assisted PSC, as an advanced two-step approach, is getting close to the champion PSC devices and could be expected great breakthrough in the future, if the heat treatment time could be reduced in the same level of one-step/two-step methods.

#### 3.1.4. Thermal Vapor Deposition

**Figure 14:**
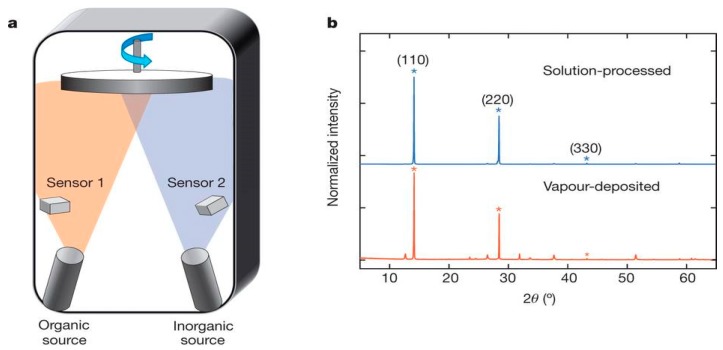
(**a**) Schematic draw of dual-source thermal evaporation and the organic source was methylammonium iodide (MAI) and PbCl_2_; (**b**) XRD spectra of a solution-processed perovskite film (blue) and vapor-deposited perovskite film (red) (Reprinted with permission) [85].

**Figure 15:**
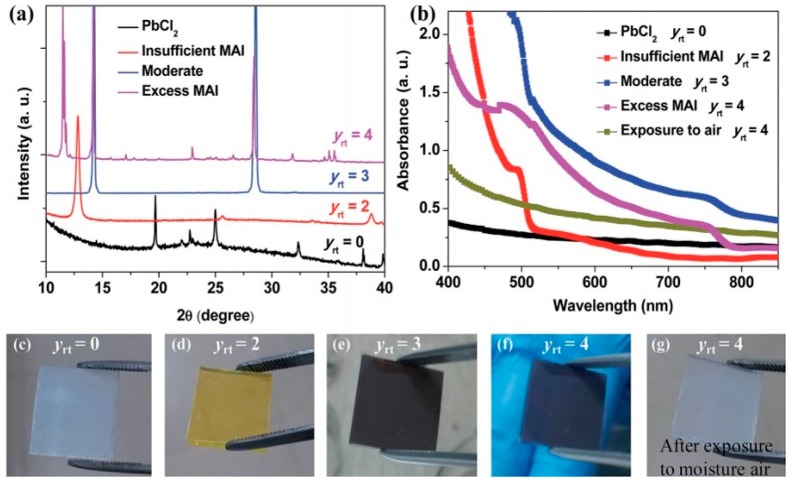
(**a**) XRD spectra, (**b**) UV-vis absorbance and (**c**–**g**) photographs of the evaporated films at different MAI/PbI_2_ ratio (Reprinted with permission) [86].

Thermal vapor deposition is among the most widely used methods in device-level thin film fabrication. The ease of source control (element/compound) and parameters such as deposition time and current/voltage guarantees film composition and surface uniformity. The first reported thermal-vapor-deposited perovskite was reported by Mitzi et al. [84]. Liu et al. [85] applied a co-evaporation with sources of MAI and PbCl_2_/PbI_2_ on rotated substrate and they fabricated a planar structure PSC of 15.4%. Figure 14 showed the evaporation system and film XRD spectra, where vacuum-deposited sample could also maintain same crystal structure after post-annealing. A further research unveiled that during co-evaporation, the reaction between PbCl_2_ and MAI tended to form PbI_2_ at first, then transferred into MAPbI_3_ under continuous MAI incorporation. Finally, the residual MAI would be found in a form of MAPbI_3_·xMAI [86]. This compositional change could be easily found out due to clear color change as shown in Figure 15. Dual-source thermal evaporation was also applied for fabricating other kinds of PSCs: Ma et al. [87] reported a CsPbIBr_2_ cell by using CsI and PbBr_2_ as evaporation sources. They acquired a PCE of 3.7% under forward scan and an efficiency of 4.7% under reverse scan. MAPbI_3_ compound source was also applied for vacuum thermal evaporation: Liang et al. [88] reported a successful MAPbI_3_ film fabrication by using their synthesized MAPbI_3_ crystals as the powder source. After a vacuum deposition under 500 W for 15 min with a 100 °C post-annealing for 20 min, they fabricated a smooth, densely-packed MAPbI_3_ thin film with great visible light absorption. However, no further device fabrication and characterization information was found. Also, those vacuum-evaporated devices rarely showed a PCE comparable with solution-based PSCs.

Although few reports agreed the thermal vapor deposition could be an effective method on PSC fabrication due to both low efficiency and extra vacuum preparation, which could increase the total cost. Thermal evaporation has its own advantages on forming fully-covered, pinhole-free films and a combination of thermal evaporation with conventional solution-based method may be expected with a better surface coverage, which equals to better device performance. Tao et al. [89] reported a PCE of 17.6% with the perovskite layer prepared by first evaporating a PbI_2_ layer and then spin-coating a MAI layer followed by annealing at 100 °C, 80 min, indicating future possibility for a hybrid fabrication approach. Besides, co-evaporated MAPbI_3_ was also sandwiched between organic charge transport layers. With a slow deposition rate of 0.5 Å/s, Malinkiewicz et al. [90] claimed a PCE of 12%. This approach was later modified by Calio et al. [91] through applying different charge transport layers above and below perovskite layer. Their best device achieved an efficiency close to 15%.

Other similar vacuum-based methods, such as layer-by-layer evaporation [92] and chemical vapor deposition [93] was also reported. Carefully monitoring evaporation profile is necessary to enhance film quality because of the low thermal stability of perovskite materials like MAPbI_3_. Due to higher complexity of those vacuum-based approach than most widely-used spin-coating, thermal vapor deposition is still not the mainstream for PSC fabrication.

### 3.2. Fabrication of Other Components in PSC

The *n*-type electron transport layer is directly relevant to the performance of PSC. The generated electron-hole pairs inside perovskite would experience charge separation at ETM/perovskite interface and form the output current. Proper ETM could also affect the growth and coverage of perovskite. A suitable ETM should have a proper band alignment: a low-enough lowest unoccupied molecular orbit (LUMO) or CBM between contact and perovskite to allow electron separation and transport, also an adequate band gap to block holes. The ETM should be also stable enough to protect the internal perovskite with HTM layer to avoid external damages especially from moisture.

Metal oxides, such as TiO_2_, are the most common used ETMs. Originated from the successful service in DSSC, TiO_2_ was the first ETM in PSC fabrication and is still used in many high-efficiency PSCs. Studies had shown TiO_2_ a perfect band structure and great electron mobility for both crystal and mesoporous structures [94]. According to different device structures, the TiO_2_ layer could be sorted as two-layer compact/mesoporous (mp) and one-layer planar structure. Mostly, the bi-layer TiO_2_ was fabricated by a sequential deposition where the dense compact layer was done by spray pyrolysis and the mp-TiO_2_ was fabricated by spin coating, which was also applied in one-layer TiO_2_ fabrication. But many alternative fabrication methods have also been demonstrated successful and corresponding results are summarized in Table 1. On considering the cost-effectiveness, most fabrication methods are non-vacuum based with a following high temperature annealing. However, TiO_2_ was also found to be responsible for UV instability of perovskite and a UV filter was suggested for further TiO_2_-based PSCs’ application [95]. Detail mechanism would be discussed later in this review. Besides, the PSCs with planar TiO_2_ were also mostly reported with a device hysteresis. Thus, more inorganic ETMs with similar band structure (ZnO, SnO_2_, BaSnO_3_, etc.) [11,17,96] are reported with great PCEs. *N*-type doping was also tried for band engineering in order to enhance the voltage potential and charge injection speed, which are directly relevant with V_oc_ and J_sc_. The current published record 21.2% PSC was fabricated with a Li-doped BaSnO_3_ (LBSO) [17]. Post-annealing treatments were also applied to improve surface morphology of ETM. Cojocaru et al. [97] reported an enhanced PCE with better TiO_2_ morphology through TiCl_4_ and UV treatment. Moreover, different ETM configurations, such as nano-rods [98], were also developed to enhance carrier transportation.

Organic ETMs, as a replacement of metal oxides, started from C_60_ and PCBM [113,116]. However, the low PCE leaded to further modifications such as n-type doping, solution engineering and interface control. Also, pairs of organic-inorganic ETL had been reported in inverted mesoporous devices and recently, some self-synthesized n-type small organic molecules also appeared in high-efficiency PSCs. At present, organic ETMs only appeared in superstrate-configured devices. Although organic ETMs had been proved to be a suitable hole blocking layer, the possible low compact between those organic ETMs and ITO/FTO substrates could be the key to this issue.

The application of graphene/graphene oxide inside the ETM also started attracting research attention. Graphene has been demonstrated with great carrier mobility and transparency, which is expected to be an enhancement inside the typical ETM layers. Relevant research progress has achieved a device efficiency of 14.5% where small portion of r-GO was mixed with mesoporous TiO_2_ ETM. The application of graphene-related component in either ETM or HTM, although still has distance with top-record PSCs, still deserved more attention and efforts [118].

The first demonstrated HTM was spiro-MeOTAD as a replacement of corrosive liquid electrolyte. This compound could be found in many top-level PSCs. Meanwhile, other HTMs with suitable electronic structure such as PEDOT: PSS [116], PTAA [102], NiO [114], CuSCN [100] were also applied in the fabrication of PSC. Among them, PTAA is becoming an excellent replacement of spiro-OMeTAD and it is already appeared in the current record device. These reported HTMs could be summarized into three categories: organic polymers, inorganic compounds and small molecules. While typical HTM fabrication approaches are spin-coating, few reports also mentioned other methods including spray [119] and sputtering [115] for inorganic HTMs. Corresponding information was listed in Table 1.

Proper doping is also a common enhancement for HTMs as in ETMs. For spiro-MeOTAD, the widely accepted dopants are the bis(tri-uoromethylsulfonyl) amine lithium salt (Li-TFSI), 4-tertbutylpyridine (TBP) and a series of organic cobalt salt such as tris(2-(1H-pyrazol-1-yl) pyridine) cobalt(III) tris(hexafluorophosphate) (FK102) and tris(2-(1H-pyrazol-1-yl)-4-tert -butylpyridine)-cobalt(III)-tris(bis(tri-fluoromethylsulfonyl) imide) (FK209) [120,121]. However, Li-TFSI would have a side effect for perovskite degradation [122]. Other methods such as modifying the molecular structure could be found in other literature [123].

At present, PSCs without ETM or HTM part had also been developed to avoid the high cost of ETM and HTM synthesis and fabrication. In those designs, the ETM or HTM was replaced by contacts with modified band structure in order to extract carriers. The perovskite layer could also be blended to possibly enhance the charge separation. Delgado et al. [124] showed an ETM-free perovskite/fullerene cell with a PCE of 14.3%. Duan et al. [125] also applied an ultra-thin graphite as the hole-extractor and acquired a PCE of 14.07%. However, those designs still had a relative weak performance due to lack of efficient carrier extractor. However, they could be helpful to understand the solar cell physics inside the perovskite layer.

## 4. Challenges

### 4.1. Cell Stability

Since PSCs has already achieved comparable performance against the Si-based PVs, the biggest challenge for PSC is to demonstrate device stability to be a suitable alternative PV technology of silicon. Reports about some long-term device characterization have been published during recent years [126,127,128,129] but most of those tests are processed in a relatively mild condition. Even under those conditions, the performance of PSCs was still not optimistic. Meanwhile, various works discovered the instability of perovskite under moisture [130], oxygen [131] and UV [132]. The perovskite materials and fabrication process also contain traps for perovskite degradation [133]. Thus, more understanding and improvement are required to upscaling of the performance of PSCs.

#### 4.1.1. Stability of Perovskite Materials

##### Crystal Structure Stability

Crystal structure and phase transition would largely affect material properties. For the ABX_3_ perovskite materials, its stability could be described with the well-known tolerance factor from Goldschmidt (1927):

(1) $$t=\frac{r_{A}+r_{0}}{\sqrt{2}(r_{B}+r_{0})}$$

where, *r_A_*, *r_B_* and *r_0_* are ionic radius for organic cation A, inorganic cation B and halide anion X, respectively. The ideal cubic perovskite structure would have a *t* = 1 and the cubic structure can only be acquired when 0.89 < *t* < 1 [6]. Lower tolerance factor means lower symmetry and the perovskite would shift to orthorhombic or tetragonal structure, which would give a negative effect on the opto-electronic properties of perovskite [134]. Most stable perovskite materials have to satisfy a 0.8 < *t* < 1 [135] and the most stable perovskite material is still MAPbI_3_, which has a tolerance factor slightly higher than 0.9 [136].

Besides the ion radius, the temperature and pressure could also affect perovskite phase transition: MAPbI_3_ was known to have a phase transition from cubic to tetragonal around 55 °C, which is within the operation temperature range of solar cells (−40 °C to 85 °C). Other researches about perovskite phase transition found that as temperature increased, the perovskite phase would transit from lower symmetry to higher symmetry (orthorhombic-tetragonal-cubic) [31,32]. Weber et al. also discovered MAPbBr_3_ and MAPbCl_3_ could maintain better symmetry than MAPbI_3_ from −40 °C to 85 °C and further details are shown in Table 2 [137]. However, PSCs solely with those two kinds of light absorbers have not shown significant high efficiency and mixed halide PSCs, even though claimed to have a better performance than MAPbI_3_, was still not comparable with the record devices. Another early study showed FAPbI_3_ had a better thermal stability. The corresponding phase transition temperature of FAPbI_3_ lied at 150 °C [138]. However, FAPbI_3_ is also reported highly unstable under moisture, which is also required during stability testing. Their work also reported a better thermal stability of an alternative MASnI_3_ and could maintain cubic phase under room temperature [139]. The pressure could also trigger perovskite phase transition, as another study claimed—as pressure increased as from 0 to 0.3 to 2.7 GPa, their MAPbI_3_ experienced phase transition of tetragonal-cubic-orthorhombic. After 4.7 GPa, the amorphous phase started appearing and lead a phase separation [140].

**Figure 16:**
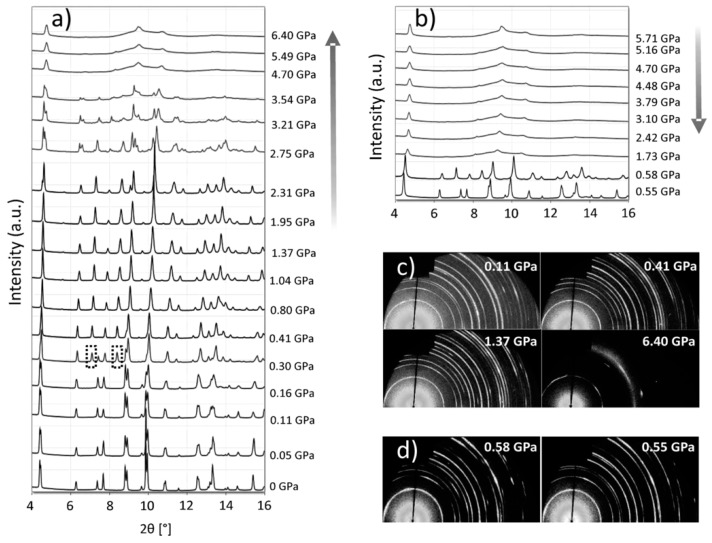
XRD patterns of MAPbI_3_ during (**a**) compression and (**b**) decompression. The highlighted by broken-lined boxes are peaks for cubic phase; (**c**,**d**) are 2D XRD patterns at specific pressures (Reprinted with permission) [140].

Figure 16 showed a clear XRD pattern change along with pressure loading and unloading. A more detailed discussion focused on lower pressure range (<200 MPa) showed pressure has much less impact on MAPbX_3_ (X=Cl, Br, I) phase transition [141]. Therefore, perovskite is expected to be suitable for normal applications without high pressure.

**Figure 17:**
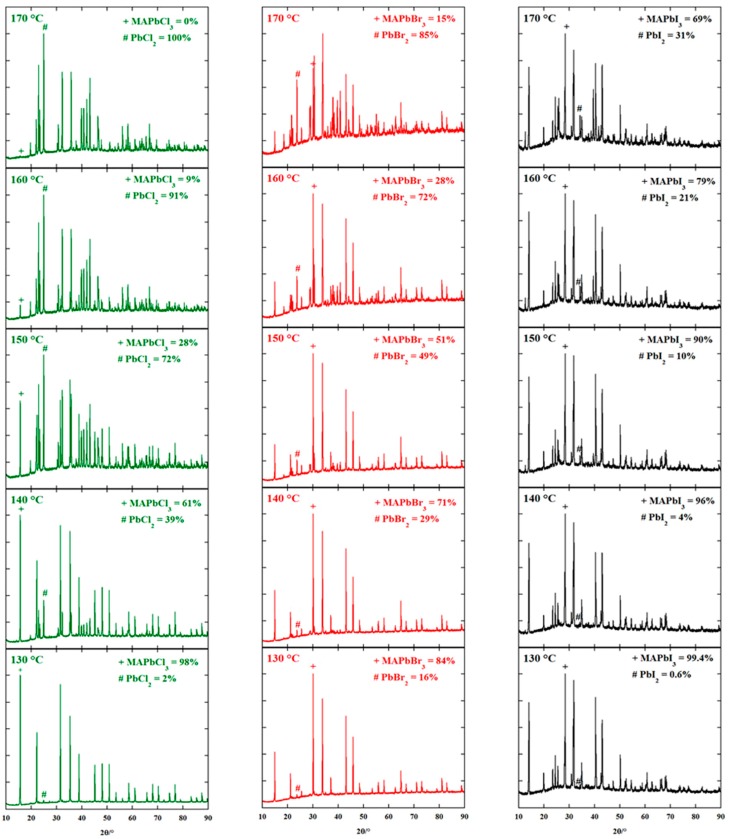
XRD patterns of MAPbX_3_ after each isotherm in the non-ambient reactor chamber (Reprinted with permission) [145].

In addition, the temperature would also lead to perovskite decomposition. Pisoni et al. [142] reported the low thermal conductivity of MAPbI_3_ and equivalent results were calculated by other groups [143,144]. Moreover, since the decomposition of MAPbX_3_ (X=Cl, Br, I) was observed starting from 130 °C as new peaks shown in XRD patterns (see Figure 17) [145], those kinds of perovskite would most likely suffer an efficiency lost during the long-term device operation due to accumulated light-generated heat inside the light absorber.

##### Environmental Stability

During device operation, the moisture, oxygen from air and high-energy photon from UV would decompose the perovskite layer within adequate time duration. A possible reaction mechanism is shown below [146]. As described in Equations (2)–(5), with adequate moisture injected into perovskite, the MAI would be dissolved in moisture and left inorganic halide. The organic-halide would continue the hydrolysis and release HI. Since HI could be continually consumed with the assistance from oxygen and photon, the decomposition is irreversible with the existence of moisture. In addition, the perovskite itself and organic cation also tend to decompose under continuous sunlight exposure (Equations (6)–(9)). However, according to those decomposition mechanism, the oxygen itself could hardly trigger the perovskite decomposition and studies also suggested perovskite samples could be stored in dry and dark environment [13]. Aging test of PSCs under white light without UV source also demonstrated acceptable device stability [75].

(2) $${\mathrm{CH}}_{3}{\mathrm{NH}}_{3}{\mathrm{PbI}}_{3}\left(s\right)\overset{H_{2}O}{\Leftrightarrow }{\mathrm{PbI}}_{2}\left(s\right)+{\mathrm{CH}}_{3}{\mathrm{NH}}_{3}I\left(\mathrm{aq}.\right)$$

(3) $${\mathrm{CH}}_{3}{\mathrm{NH}}_{3}I\left(\mathrm{aq}.\right)\overset{}{\Leftrightarrow }{\mathrm{CH}}_{3}{\mathrm{NH}}_{2}\left(\mathrm{aq}.\right)+\mathrm{HI}\left(\mathrm{aq}.\right)$$

(4) $$4\mathrm{HI}\left(\mathrm{aq}.\right)+O_{2}\overset{}{\Leftrightarrow }2I_{2}\left(s\right)+2H_{2}O\left(l\right)\;$$

(5) $$2\mathrm{HI}\left(\mathrm{aq}.\right)\overset{}{\Leftrightarrow }H_{2}\left(g\right)+I_{2}\left(s\right)$$

(6) $${\mathrm{CH}}_{3}{\mathrm{NH}}_{3}{\mathrm{PbI}}_{3}\left(s\right)\overset{h\nu }{\Leftrightarrow }{\mathrm{PbI}}_{2}\left(s\right)+{\mathrm{CH}}_{3}{\mathrm{NH}}_{2}↑+\mathrm{HI}↑$$

(7) $$2I^{-}\overset{}{\Leftrightarrow }I_{2}+2e^{-}$$

(8) $$3{\mathrm{CH}}_{3}{\mathrm{NH}}_{3}^{+}\overset{h\nu }{\Leftrightarrow }3{\mathrm{CH}}_{3}{\mathrm{NH}}_{2}↑+3H^{+}$$

(9) $$I^{-}+I_{2}+3H^{+}+2e^{-}\overset{}{\Leftrightarrow }3\mathrm{HI}↑$$

Studies have shown the relations between air humidity and perovskite decomposition: Kelly et al. [147] showed a positive correlation between humidity and PCE: as relative humidity (RH) increased from 50% to 80% in N_2_, the absorption at 410 nm drastically decreased. Their work also proved proper HTM layer could reduce the moisture invasion rate by maintaining a good coverage. However, such effect could only last few hours and later, the absorption would continue fast decreasing. Moreover, Han et al. [148] showed the perovskite degradation could be more severe with the corporation of humidity and temperature: as shown in Figure 17, the device PCE would be almost zeroed under AM 1.5G within 20 h under high temperature (55 °C in air and 85 °C for internal device temperature) and RH (80%).

Due to such side effects from oxygen, UV and mostly moisture, the preparation of perovskite was mostly processed inside the glove box. However, some studies showed that proper relative humidity. According to Gangishetty et al. [149], higher RH could be helpful on enlarging perovskite crystal sizes and better connections among crystals during two-step fabrication. A later study displayed a possible best combination of ambient humidity and annealing time during perovskite fabrication: Their best device was fabricated under 20% RH and 45 min post-annealing [150]. However, although the humidity-incorporated synthesis process had successfully produced a planar PSC with 19.3% PCE [151]. The moisture offered an extra solubility for organic precursors but also leaded a PCE decrease to less than 5% after few days under ambient atmosphere.

**Figure 18:**
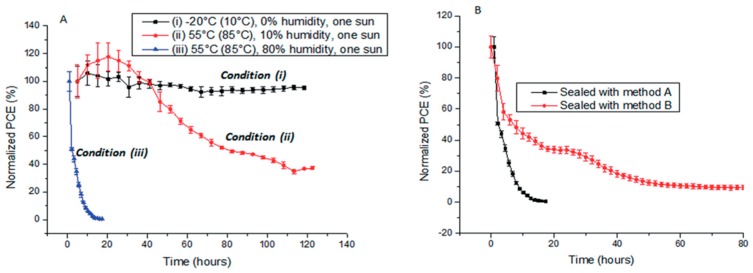
(**A**) Stability testing of PSCs sealed by method A under three different environmental conditions; (**B**) comparison of the stability of devices sealed by method A and B and tested under environmental condition (iii). All PCEs are already normalized (Reprinted with permission) [148].

**Figure 19:**
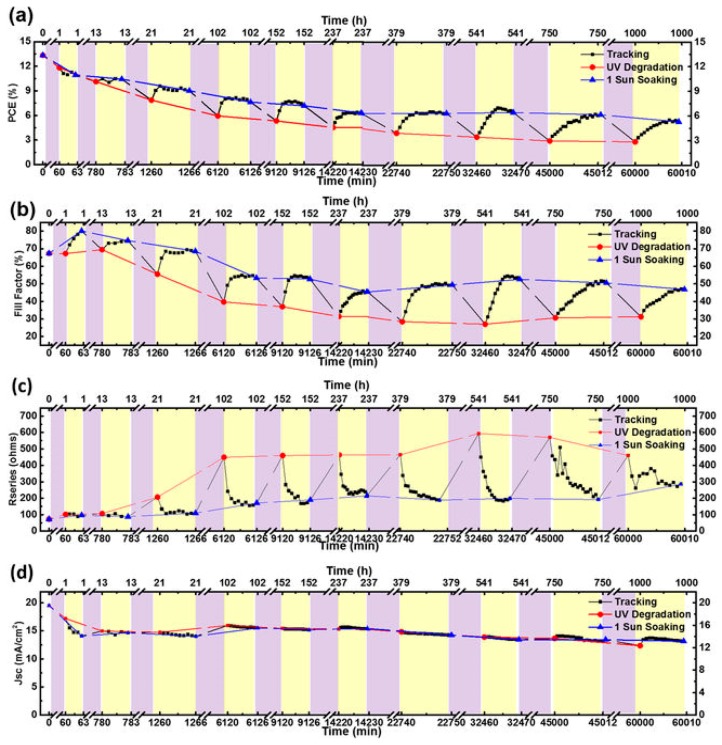
UV degradation/recovery cycle of (**a**) PCE; (**b**) FF; (**c**) R_s_ and (**d**) J_sc_ for device subjected to a range of UV exposure and 1-sun illumination. Purple regions represent UV exposure and yellow regions represent 1-sun light illumination periods (Reprinted with permission) [153].

The degradation of perovskites by UV light could be originated at the TiO_2_ layer. With assistance from UV light, TiO_2_ could interact with I^−^ and form I_2_ as in typical DSSCs. Therefore, it could destroy the perovskite crystal structure and strengthen the ionic reaction process of organic cations [152]. Moreover, a further study on UV degradation mechanism found with UV-AM 1.5G illumination cycle could partially recover the device performance. It is the hole traps accumulated on the perovskite/TiO_2_ interface and flowed into other charge transport layer due to insufficient charge neutralization [153]. Those traps were also reasons of lower J_sc_ during following AM 1.5G illumination. Figure 18 and Figure 19 displayed their UV-1 sun cycling illumination test results and possible mechanism. Thus, several methods had been tried to separate perovskite and TiO_2_. Besides the Sb_2_S_3_ inserting reported by Ito et al. [152]. Applying a UV-filter on the TCO substrate before TiO_2_ deposition and several reports also showed the stability improvement. However, compared with other two approaches, the UV-filter might trigger an unavoidable fabrication cost increasing due to extra materials cost. Since PSCs have to pass the more important aging tests under high temperature and high RH in order to pass the stability standards of thin film PV, the UV problem is currently not owing high priority.

Recently, the application of graphene and its relevant oxides in the PSCs has attracted attentions. Experiments already demonstrated that graphene, due to its outstanding carrier mobility and high transparency, could be able to enhance the device performance of PSCs [118]. The contribution of graphene to PSCs, however, could also contain the device stability and the extension of the lifetime of perovskite. Graphene/graphene-oxides could replace the HTM/ETM layer or inserted between perovskite and other HTM/ETM or between HTM/ETM and metal contacts. Due to the wetting transparency of 2D graphene [154], the decomposition of perovskite could be released. The small lattice size of 2D graphene could also resist the inter-diffusion of metal ions from either perovskite or metal contact [155]. Agresti et al. investigated the effect of graphene and graphene oxide [156] and their results indicated that the graphene oxide would join the perovskite decomposition reactions at high intensities. The graphene added inside the mesoporous TiO_2_, however, could improve the device stability under both dark and continuous illumination environment due to faster carrier transportation. They also suggested that PSCs with graphene/graphene oxide would suffer an efficiency and J_sc_ loss under prolonged thermal stress. Doped graphene could be also suitable for increasing the stability of PSCs. Bi et al. [157] reported a stable PSCs with its PCBM layer mixed with n-type graphene. The device efficiency was stable for 500 h at 85 °C. Thus, it can be expected that, due to excellent electrical properties of graphene, new contacts based on graphene rather than metals could be considered. Therefore, both device stability and efficiency might be improved.

Graphene is not the only 2D material that helps improving the stability of PSCs. Other materials that can be prepared by the mechanical exfoliation method has been also tried in PSCs to increase the device stability and efficiency. One investigation applied a combined structure of graphene/MoS_2_ at the interface of PCBM/Ag. The addition of this mixed interlayer increased the parameters of PSC as well as the stability of J_sc_, V_oc_ and PCE for the initial several hours [158]. Chen et al. even fabricated the typical carrier transport material, TiO_2_, into a 2D structure (2D atomic sheets of titania) [159]. Their results showed that the PCE of their PSCs could be comparable with the standard PSCs by optimizing the number of 2D-TiO_2_ layers. This new structure of TiO_2_, according to their investigation, could also reduce the UV absorption, which is one of the key to the decomposition of perovskite.

#### 4.1.2. Stability of Other Components

**Figure 20:**
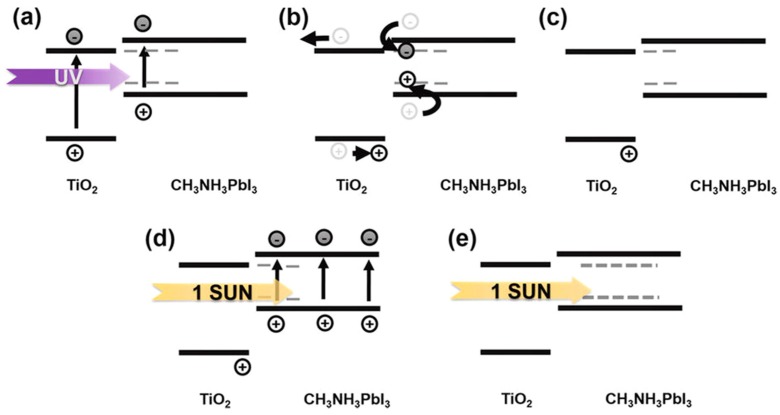
Schematic draw of the proposed mechanisms for UV degradation and recovery of PSCs (Reprinted with permission) [153].

Low stability of other components (HTM, electrodes and etc.), like lower-quality perovskite, would also largely minimized the device performance of PSC. Since most developed HTM candidates are organic compounds, such as the most typical spiro-OMeTAD based HTM series, the temperature control becomes a vital factor. A detailed report form Wu et al. [160] showed that, although low temperature annealing could enhance the formation and crystallization of spiro-OMeTAD, the transfer of additive Li-TFSI to the TiO_2_ surface and the evaporation of 4-tert-butylpyridine (TBP), also another common additive, would both compress the device voltage potential by changing fermi level of TiO_2_. Therefore, the device acquired lower V_oc_. In addition, spiro-OMeTAD, as discussed by Kelly et al. [147], may suffer from cracking during the fabrication process. Thus, the internal perovskite would be easily exposed and device degradation may be accelerated. The mechanism of UV degradation and recovery of PSCs is given in Figure 20 [153].

As a consequence, other stable HTM layers including o,p-dimethoxyphenyl-based biphenyl (HL-1) and carbazole (HL-2) [161], single-walled carbon nanotube (SWCNT) enhancement [162], tetrathiafulvalene derivative (TTF-1) [96], poly(3-hexylthiophene) (P3HT) [163] had been developed and literatures showed great progress on device stability. Recently, a French group reported a stable PSC with CuSCN inserted between Spiro-OMeTAD and gold electrode. The device efficiency only lost 5% after 1000 h running under 60 °C in nitrogen atmosphere. Another test under more realistic environment (85 °C, 1000 h, air, dark) showed a 15% efficiency loss. The thin layer of CuSCN was believed to block the metal diffusion, which is the reason for device degradation [164]. Although more realistic testing of durability (e.g., 85 °C, 1000 h and high humidity) is still necessary before the industrial application of perovskite, this result showed a noteworthy progress of the stability of PSCs. In addition, a hydrophobic HTM could be another plus due to the irreversible perovskite corrosion from the moisture.

### 4.2. J-V Hysteresis

Another barrier for PSC’s further development is the J-V hysteresis, which was observed when applying different voltage sweeping rates and directions [165]. The best efficiency results are usually acquired at V_oc_ rather than J_sc_, P_max_ or under reverse bias condition. Two major categories of hysteresis have been found: normal and invert hysteresis. The normal hysteresis leads to a higher efficiency during reversed bias scan (voltage decreases) but lower efficiency during the opposite scan. The inverted hysteresis goes exactly the opposite way. These two kinds of hysteresis could exist together or appear separately depending on the applied pre-poling bias [166]. Different voltage sweeping rate would also change efficiency results and these changes of device parameters are mostly random. Such phenomenon is also not relevant with device structure. Therefore, rather than other kind of PV technology, standardizing PSC measurement becomes a challenging task and even those reported progress, including both PCE and device stability breakthrough, might become questionable. Although recent reports claimed that their devices displayed a low or little J-V hysteresis during PCE measurements [167,168] although this J-V hysteresis is still noticeable during most of the PSC characterizations.

The mechanism of hysteresis is still unknown but several hypotheses had been established. Ferroelectric polarization [169], ion migration [170], charge trapping [171] and capacitive effects [172]. Several reviews already offered intensive discussion about those hypothesis [173,174]. Recent research starts to support that both ion migration and charge trapping could the reasons for the J-V hysteresis and relevant detail discussion can be found elsewhere [175]. Since J-V hysteresis had such negative effect, improved PCE measurements technique was suggested [176,177].

## 5. Toxicity

The toxicity of perovskite comes from the widely-used lead inside MAPbI_3_ and environmental concerns would be appeared especially on the issue of large-scale fabrication waste treatment. Although calculations already showed the possible contamination from perovskite would be relatively insignificant compared with other lead pollutions [178] and the production of PSC could be able to use waste lead from daily waste [179], studies on lead-free PSCs cannot be neglected. Tin was the first well-studied replacement metal cation since Sn and Pb are both carbon periodic elements, thus, MASnI_3_ is believed to be able to maintain the same crystal structure as MAPbI_3_. The fact, as shown by Noel et al. [180], is that Sn^2+^ could be easily oxidized to Sn^4+^, leading a weak device performance. Other trials of introducing organic/inorganic additives to retard tin oxidation had also been reported [181,182] but their device PCE was still not promising.

Due to this chemical instability of pure tin-based perovskite materials, the hybrid Sn-Pb metal cations in perovskite could be more realistic and the more advanced PCE also demonstrated this idea: Zhu et al. [183] reported a remarkable PCE of 15.2% with a light absorber of MASn_0.25_Pb_0.75_I_3_ and a suitable control of DMSO additive and a PCBM:C_60_ electron transport layer. Another study also indicated MASn_1-x_Pb_x_I_3_ could have an electronic structure closer to MASnI_3_ than MAPbI_3_ even with few Sn replacement [184]. All those results indicated that from the view of reducing process toxicity, tin is not a perfect candidate to totally replace lead due to its chemical instability.

**Figure 21:**
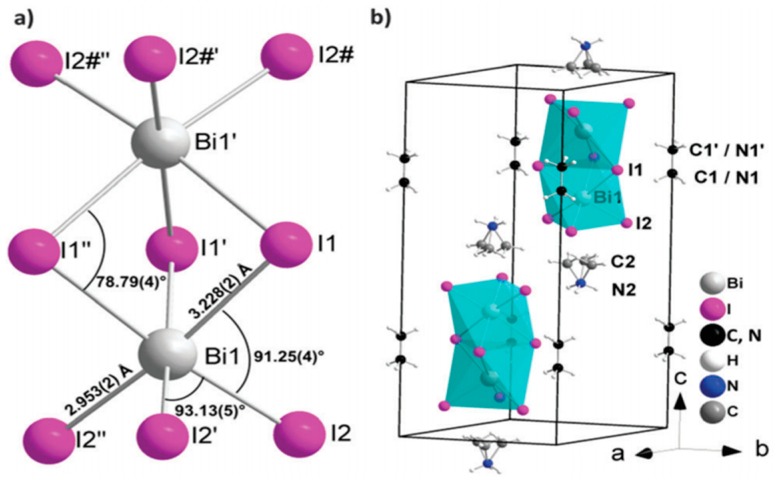
Crystal structure of (CH_3_NH_3_)_3_Bi_2_I_9_ (MBI): (**a**) local structure of the (Bi_2_I_9_)^3−^ anion; (**b**) cation and anion positions in the unit cell (Reprinted with permission) [185].

Another intensive-studied candidate is the neighbor of lead: bismuth. Bi could form a stable (MA)_3_Bi_3_I_9_ (MABI) perovskite material. Its crystal structure was shown in Figure 21 [185]. Similar as Sn-doped MAPbI_3_, MABI also showed better stability under ambient air for 1000 h [186]. The first reported MABI-based perovskite only reached a low efficiency of 0.12% with a relatively low V_oc_ of 0.68 V and an extremely low J_sc_ of 0.52 mA/cm^2^ [187]. At present, the Bi-based perovskite could only able to reach an efficiency of 0.42% due to low J_sc_ [188]. A recent study investigated the absorption and recombination dynamics of excitations inside the MABI crystals: the I(5p)-Bi(6p), I(6p) excitation [189] is localized in (BI_3_)^-^ units, resulting little free carriers released at the MABI/TiO_2_ interface [190]. Therefore, they suggested considering bulk-heterojunction structure with nano-scale MABI crystals in order to possibly enhance the low J_sc_. Some other reports focused on Bi-based halide double perovskites such as Cs_2_AgBiX_6_ (X=Br, Cl) [191] and (MA)_2_KBiCl_6_ [192]. But no effective devices had been reported and deep understanding of optoelectronic properties are still suggested. Thus, Bi-based PSC is still not promising at present, even compared with Sn.

Other types of lead-free perovskites such as CsGeI_3_ [193], MAGeX_3_ (X: Cl, Br, I) [194], MASrI_3_ [195], MACaI_3_ [196] had also been reported but those materials are either not suitable for visible light absorption due to large band gap [195,196], or only showed low efficiency of less than 1%. Most of the material characterizations are still in lack. Thus, non-toxic perovskite development and corresponding fabrication of PSCs still has a long way before replacing the position of lead.

## 6. Discussion and Future Research Efforts

The above research efforts indicated that the PSCs will have a greater potential for commercialization if the stability of cells can be improved. The high efficiency and low-cost manufacturability to harvest terawatt levels by solar energy are very attractive with this next generation solar cell technology. The cell degradability is identified as due to primarily the exposure of perovskite layer to water vapor and heating effects, which change the active phase of lead-based perovskites. There are numerous efforts to improve the stability of these solar cells by many groups worldwide. Development of perovskite layers using other metals has been tried with poor success to address the toxic issue and stabilizing the perovskite structures. Also, cell passivation has been investigated to stabilizing the cells by prevention of perovskite layer to the ambient. Another approach is to reduce the heating effect by utilizing IR absorbance layers or external components. Also, integration of few of these technologies may improve the stability of this solar cell technology from current stability records of around six months.

Also, it is important to address the harmfulness of lead-based compounds in PSCs. While development of other metal-based perovskite is also interested in the viewpoint of environmental protection, their effectiveness does not reach the efficiency of lead-based compounds. Furthermore, better recycling methodologies are important to prevent the transfer of lead compounds into the environments. Similar environmental issues have been addressed for CdTe solar cells and thus, it is possible to utilize already existing infrastructure for recycling and environmental protection issues. The environmental protection authority regulates the lead content of drinking water below 0.015 g/L. Authors believe that these areas can be further improved by research efforts.

It is also important to address that, due to the transparent nature of some of the HTM/ETM and the electrodes, the PSCs could be fabricated with a structure that could absorb sunlight from both directions. An investigation in 2016 already found out that, with the help of transparent solution-processed silver nanowires (AgNWs), an efficiency of more than 11% and 7.53% could be observed with front and back illumination, respectively [197]. Together with passivation of PCSs using transparent insulators such as polymers, the cell performance as well as durability may be enhanced. Thus, investigation on the bi-facial PSCs could also be another research direction for the improvements of PSCs.

## 7. Conclusions

The PSC has experienced a significant improvement from 3.8% to 22.1% since 2012 and the perovskite-based tandem cell has already achieved 26.7%, creating a new record in history of PV technology. Numerous research efforts on both PSC efficiency improvements and deeper understanding about perovskite materials’ outstanding electrical and optical properties, such as largely-tunable band gaps for light absorption, high absorption coefficients, large carrier diffusion lengths, great carrier mobility, have been established during the past few years. The current PSCs already combined structural advantages of both DSSCs and thin film PV since the discovery of perovskite and become a new challenger for Si-based PV dominant market share, not only due to record 22.1% efficiency for small area but also comparable larger-area device efficiency. The vast discovery and successful application of organic/inorganic charge transport materials and blocking layers also assisted the formation and crystallization of perovskites and helped charge transfers at the interfaces. Many kinds of PSC fabrication approaches have also been developed and most of them could fall into four major categories: one-step; two-step; vapor-assisted solution method; and thermal vapor deposition with a top PCE of 22.1% (current record), 20.26%, 16.48% and 17.6%, respectively. Also, numerous hybrid perovskite fabrication process was also invented, which is uncommon for other types of PVs. According to current progress, it is reasonable that the next high-efficiency PSC may be still based on solution-based approaches (e.g., spin coating) with a mixed perovskite phase, as applied in the record 22.1% and the stable 21.2% devices.

The PSCs still have great barriers for further improvements. The biggest problem comes with the natural instability of perovskite materials, especially the most widely-used MAPbI_3_. The phase transition within the range of solar cell operation temperature brought problems on device usage. The instability with varying temperature and pressure leads extra concerns for device fabrication. The moisture, UV light and oxygen would also bring irreversible damage to the perovskite layers, which largely reduced the device stability and commercialization of PSC. The efforts such as elemental adjusting, device sealing and extra blocking layer inside the device had been tried to solve these problems but more stability tests under harsh environments are strongly suggested for PSCs to reach the required standard.

Other drawbacks such as J-V hysteresis and toxicity of lead made it difficult to further improve the performance of PSCs. While the mechanism of hysteresis was still inconclusive, the lead toxic has attracted many research efforts on considering the non-toxic replacement pf perovskite materials. Research work found out that all candidates, from the neighborhood Sn, Bi and new candidates as Cs, Ge, suffered a great loss of J_sc_, which directly leads to the huge loss in PCE. A more complicated replacement profile might be the solution of lead-free PSCs. Although efforts claimed low hysteresis in some PSCs, deep theoretical understanding and standardized testing protocol is suggested for PSCs.

Perovskite, compared with other PV techniques (thin film, organic, dye-sensitized), could be the best alternative solar absorber. As efforts on better perovskite layer formation and longer device durability, even the lead-based PSCs could be able to share a certain part of PV market. Such trend could influence further research and development (R&D) efforts towards higher-stable and non-toxic devices.

## Figures and Tables

**Table 1:** A summary of PSCs’ performance with different ETM/HTM pairs, fabrication methods were also labelled. All materials without labelling were spin-coated.

ETM	HTM	J_sc_ (mA/cm^2^)	V_oc_ (V)	FF	η (%)	Ref.
c-TiO_2_/mp-TiO_2_ (spray pyrolysis)	spiro-MeOTAD	22.3	1.06	0.77	18.1	[99]
	CuSCN	21.8	1.1	0.692	16.6	[100]
	FDT	22.7	1.15	0.76	20.2	[101]
	PTAA	20.7	1.05	0.74	16.1	[102]
TiO_2_ (spray pyrolysis)	CuGaO_2_	21.66	1.11	0.77	18.51	[103]
TiO_2_ (spray pyrolysis)	PTAA	20.1	1.1	0.78	17.2	[104]
TiO_2_	spiro-MeOTAD	21.5	1.07	0.67	15.4	[105]
TiO_2_	P3HT	19.1	0.98	0.66	12.4	[106]
TiO_2_ (with screen printing, C_60_ treatment)	spiro-MeOTAD	19.6	0.84	0.72	11.7	[98]
TiO_2_ (ALD)	Carbon	19.53	0.965	0.4147	7.82	[107]
TiO_2_ (ALD)/PCBM	NiOx	19.7	0.93	0.477	8.8	[108]
TiO_2_ (sputtering)	PTAA	20.77	1.108	0.69	15.88	[109]
ZrO_2_/TiO_2_	spiro-MeOTAD	22.20	1.05	0.8	17.88	[110]
TiO_2_/BaTiO_3_ (TiCl_4_-treated)	spiro-MeOTAD	19.3	0.962	0.67	12.4	[111]
ZnO	spiro-MeOTAD	20.5	1.03	0.748	15.7	[11]
ZnO	P3HT	14.94	0.9329	0.6267	8.77	[112]
SnO_2_	spiro-OMeTAD	21.65	1.06	0.659	15.13	[96]
Nb-SnO_2_		22.36	1.08	0.727	17.57	
La-BaSnO_3_	PTAA	23.4	1.12	0.813	21.3	[17]
PCBM (vacuum evaporation)	NiO (sputtering)	19.8	0.96	0.61	11.6	[113]
PCBM	NiO	18.74	1.04	0.689	13.43	[114]
	NiO (sputtering)	20.33	1.08	0.69	15.15	[115]
	PEDOT: PSS	18.9	0.972	0.8016	15.32	[116]
C_60_ (vacuum evaporation)	PTAA	22.96	1.11	N/A	19.5	[117]
TiO_2_/r-GO	Spiro-OMeTAD	22.0	0.93	0.707	14.5	[118]

**Table 2:** Phase transition points of MAPbX_3_ (X=CI, Br, I) [137].

Phase	Temperature (K)	Crystal System	Space Group	Lattice (pm)	Volume (10^4^ pm^3^)
CH_3_NH_3_PbCl_3_^-^					
α	>178.8	cubic	Pm3m	a = 567.5	182.8
β	172.9–178.8	tetragonal	P4/mmm	a = 565.6	180.1
				c = 563.0	
γ	<172.9	orthorhombic	P222_1_	a = 567.3	357.0
				b = 562.8	
				c = 1118.2	
CH_3_NH_3_PbBr_3_^-^					
α	>236.9	cubic	Pm3m	a = 590.1(1)	206.3 (260 K)
β	155.1–236.9	tetragonal	I4/mcm	a = 832.2(2)	819.4
				c = 1183.2(7)	
γ	149.5–155.1	tetragonal	P4/mcm	a = 589.4(2)	
				c = 586.1(2)	
δ	<149.5	orthorhombic	Pna2_1_	a = 797.9(1)	811.1
				b = 858.0(2)	
				c = 1184.9(2)	
CH_3_NH_3_PbI_3_^-^					
α	>327.4	cubic	Pm3m	a = 632.85(4)	253.5
β	162.2–327.4	tetragonal	I4/mcm	a = 885.5(6)	992.6
				c = 1265.9(8)	
γ	<162.2	orthorhombic	Pna2_1_	a = 886.1(2)	959.5
				b = 858.1(2)	
				c = 1262.0(3)	
